# Long-term weight change and breast cancer risk: the European prospective investigation into cancer and nutrition (EPIC)

**DOI:** 10.1038/sj.bjc.6602763

**Published:** 2005-08-30

**Authors:** P H Lahmann, M Schulz, K Hoffmann, H Boeing, A Tjønneland, A Olsen, K Overvad, T J Key, N E Allen, K-T Khaw, S Bingham, G Berglund, E Wirfält, F Berrino, V Krogh, A Trichopoulou, P Lagiou, D Trichopoulos, R Kaaks, E Riboli

**Affiliations:** 1 Department of Epidemiology, German Institute of Human Nutrition Potsdam-Rehbrücke, Arthur-Scheunert-Allee 114-116, Nuthetal 14558, Germany; 2 Institute of Cancer Epidemiology, Danish Cancer Society, Copenhagen, Denmark; 3 Department of Clinical Epidemiology, Aalborg Hospital and Aarhus University Hospital, Aalborg, Denmark; 4 Cancer Research UK, Epidemiology Unit, University Oxford, Oxford, UK; 5 Department of Public Health and Primary Care, School of Clinical Medicine, University of Cambridge, Cambridge, UK; 6 MRC Dunn Human Nutrition Unit, Cambridge, UK; 7 Department of Medicine, Lund University, Malmö University Hospital, Malmö, Sweden; 8 Epidemiology Unit, National Cancer Institute, Milan, Italy; 9 Department of Hygiene and Epidemiology, School of Medicine, University of Athens, Athens, Greece; 10 Department of Epidemiology, Havard School of Public Health, Boston, MA, USA; 11 International Agency for Research on Cancer (IARC-WHO), Lyon, France

**Keywords:** breast neoplasm, weight gain, weight history, obesity, hormone replacement therapy, menopausal status

## Abstract

We examined prospectively the association between weight change during adulthood and breast cancer risk, using data on 1358 incident cases that developed during 5.8 years of follow-up among 40 429 premenopausal and 57 923 postmenopausal women from six European countries, taking part in the European prospective investigation into cancer and nutrition study. Multivariate Cox regression models were used to calculate hazard ratios according to weight change (kg), defined as the weight difference between age at enrolment and age 20 adjusted for other risk factors. Changes in weight were not associated with premenopausal breast cancer risk. In postmenopausal women, weight gain was positively associated with breast cancer risk only among noncurrent hormone replacement therapy (HRT) users (*P*-trend ⩽0.0002). Compared to women with a stable weight (±2 kg), the relative risk for women who gained 15–20 kg was 1.50 (95% confidence interval (CI) 1.06–2.13). The pooled RR per weight gain increment of 5 kg was 1.08 (95% CI 1.04–1.12). Weight gain was not associated with breast cancer risk in current HRT users, although, overall, these women experienced a much higher risk of breast cancer compared with nonusers. Our findings suggest that large adult weight gain was a significant predictor of breast cancer in postmenopausal women not taking exogenous hormones.

Weight gain during adult life, body weight, and fat distribution may separately or in combination play a role in the development and prognosis of breast cancer. The association between weight change and breast cancer risk is modified by menopausal status, with higher weight gain associated with decreased risk for premenopausal women and increased risk for postmenopausal women (IARC working group, 2002). More specifically, for postmenopausal breast cancer risk, the most consistent body size predictor appears to be weight gain, even in studies in which general adiposity was only weakly associated with risk (Ziegler, 1997; Ballard-Barbash, 1999; Friedenreich, 2001; Willett, 2001). Adult weight change is considered a dynamic body measure unlike static measures such as body mass index (BMI), and has been hypothesised to better reflect age-related metabolic changes that may be important in breast cancer development (Ballard-Barbash, 1999). Relative risks between 1.2 and 2.3 for the highest *vs* lowest categories for weight gained between age 18 or 20 and the reference age were reported in previous case–control and cohort studies (Friedenreich, 2001; IARC working group, 2002). However, the data on weight change and breast cancer risk from prospective studies are sparse, especially among premenopausal women. Moreover, the potential effect modification by body size at young adult age (Barnes-Josiah *et al*, 1995; Huang *et al*, 1997; Magnusson *et al*, 1998; Weiderpass *et al*, 2004) or hormone replacement therapy (HRT) use (Huang *et al*, 1997; Friedenreich *et al*, 2002; Morimoto *et al*, 2002; Lahmann *et al*, 2003; Feigelson *et al*, 2004) on the association between weight change and breast cancer risk has been only investigated in a limited number of studies.

The purpose of this study was to examine the association between long-term adult weight change and risk of breast cancer in pre- and postmenopausal women, controlling for other known risk factors in female study participants from the European Prospective Investigation into Cancer and Nutrition (EPIC). A secondary aim of this study was to evaluate whether the association between weight change and risk of breast cancer is modified by (a) initial weight or BMI at age 20, and (b) current HRT use among postmenopausal women.

## MATERIALS AND METHODS

The EPIC is a multi-centre prospective cohort study designed primarily to investigate the association between nutrition and cancer. The EPIC cohort consists of 23 subcohorts in 10 European countries: Denmark, France, Germany, Greece, Italy, The Netherlands, Norway, Spain, Sweden, and the United Kingdom, thereby allowing comparisons of lifestyle and food habits among regions with very different cancer rates. Food-related and lifestyle questionnaires were administered and anthropometric measurements obtained from all participants at the time of enrolment (1992–2000). The 366 521 eligible female participants were mostly aged 25–70 years and recruited from the general population residing in a given geographical area, such as a town or a province (Riboli *et al*, 2002). Exceptions were the French cohort (health insurance scheme for school employees), the Utrecht cohort in the Netherlands (breast cancer screening attendees), the Ragusa cohort in Italy (blood donors and their spouses), and the Oxford cohort in the UK (based on vegetarian volunteers and healthy eaters). Eligible subjects were invited to participate in the study, and those who accepted gave informed consent and completed questionnaires on their diet, lifestyle and medical history. Study subjects were then invited to a centre to provide a blood sample and to have anthropometric measurements taken, methods of which have been reported in full elsewhere (Riboli and Kaaks, 1997; Riboli *et al*, 2002).

### Study population

The present study was based on data from 345 365 female participants after excluding women with prevalent cancer at any site at baseline examination, and those with missing nondietary questionnaire data. The study population was further restricted to 125 233 women from Denmark, Germany (Potsdam), Greece, Italy (Varese), Sweden (Malmö), and the United Kingdom, with available data on recalled body weight at age 20 or 25 years, and measured or predicted (Oxford, ‘health conscious group’) anthropometric characteristics at study enrolment. Women were classified according to menopausal status at enrolment based on an algorithm as described elsewhere (Lahmann *et al*, 2004). Accordingly, 33.1% of women were classified as premenopausal and 47.7% were naturally postmenopausal. Women who were perimenopausal (11.4%) or had a surgical menopause or uncertain menopausal status (7.8%) were excluded from the present analysis. Subjects with missing data on measured anthropometric characteristics at enrolment and recalled weight at age 20, and women aged >80 years at baseline were also excluded. The analytical cohort for this study therefore consisted of 98 352 women from six countries, 40 429 of whom were premenopausal and 57 923 of whom were naturally postmenopausal.

### End points and ascertainment of cases

Incident breast cancer cases were identified through population cancer registries (Denmark, Italy, Sweden, United Kingdom) or by active follow-up (Germany, Greece), depending on the follow-up systems in each of the participating countries. The active follow-up procedure used a combination of methods including health insurance records, cancer and pathology registries, and via contact with participants and their next-of-kin. Women were followed from study entry (1992–2000) until first breast cancer diagnosis, death, emigration, or end of the follow-up period. By April 2004, 5984 breast cancer cases (invasive *n*=5386; *in situ n*=593; unspecified *n*=4; metastatic *n*=1) had been reported to the common database at the International Agency for Research on Cancer (IARC), Lyon, based on information on complete follow-up data up till December 2001 or December 2002 in most of the centres. Mortality data were coded according to the 10th Revision of the International Statistical Classification of Diseases, Injuries and Causes of Death (ICD-10), and cancer incidence data were coded according to ICD-O-2. This analysis included 1358 invasive (primary, malignant) breast cancer cases, of which 264 occurred in women who were premenopausal at recruitment and 1094 in postmenopausal women.

### Classification of body measures and other predictor variables

Data on recalled weight at age 20 were available for cohorts in Italy (Varese), United Kingdom, Greece, Sweden (Malmö) and Denmark, and for the age of 25 years in Germany (Potsdam). Weight and height at recruitment were measured to the nearest 0.1 kg and 0.1 or 0.5 cm, respectively, with subjects wearing no shoes.

For the present study, baseline weight was adjusted to reduce heterogeneity due to protocol differences in clothing worn during measurement (Haftenberger *et al*, 2002). For the ‘health conscious group’ in Oxford (UK), linear regression models were used to predict sex- and age-specific values from women from the Oxford general population group with both measured and self-reported body measures (Haftenberger *et al*, 2002; Spencer *et al*, 2002). Body mass index was calculated as weight in kg divided by height in meters squared (kg m^−2^). Subjects with a BMI of ⩾25.0 to <30.0 kg m^−2^ were classified as overweight and those with a BMI of ⩾30.0 kg m^−2^ were classified as obese (World Health Organization, 1998). Adult weight change was calculated as the difference between measured baseline weight at enrolment and recalled weight at age 20 or 25 years.

Information on reproductive, sociodemographic, and lifestyle characteristics was obtained from the standardised health questionnaire at study entry. Other known risk factors included in this analysis were: age at menarche (⩽11, 12, 13, 14, >15 years), age at first pregnancy (first birth <20, 20–30, >30 years, nulliparous), education (none/primary school, technical/professional school, secondary school, university), smoking status (never, former, current), alcohol consumption as ethanol in g day^−1^ (abstainers, 1–14, 15–30, >30 g), leisure physical activity (continuous score), height (continuous), current OC use (no/yes), and current hormone use (no/yes). Current hormone use refers to the use of menopausal hormones at the time of recruitment as derived from the country-specific questionnaires or during interviews, and includes oestrogen alone and combined oestrogen/progestin preparations (referred to as HRT use).

### Statistical analysis

Cox proportional hazards models were used to estimate adjusted hazard ratios (HR) and 95% confidence intervals (CI) of breast cancer incidence for each weight change category. Weight changes were defined as follows: −2 and +2 kg as the referent category (stable weight); more than −2 kg as weight loss; and more than +2 kg as weight gain. The latter was further divided into five weight gain categories (2.1–5.0, 5.1–10.0, 10.1–15.0, 15.1–20.0, >20 kg). Age was used as the underlying (primary dependent) time variable in the counting process formulation, with entry time *t*_0_ defined as the subject's age at recruitment, and exit time *t*_1_ defined as the subject's age at breast cancer diagnosis or censoring date. All multivariate models were stratified (option ‘strata’ in SAS-PHREG procedure) by age at recruitment and by study centre to be less sensitive against violations of the proportional hazards assumption, and simultaneously adjusted for the following established or potential breast cancer risk factors: weight at age 20, height, leisure physical activity (as continuous variables), age at menarche, parity, age at first birth, education, smoking status, alcohol consumption, current OC use and current HRT use. An indicator category for missing responses for each covariate was created to minimise the loss of observations due to missing covariate data.

Trend tests were calculated on the basis of category-based scores, assigning a score from 1 to 7 to an individual according to the weight change categories. For country-specific analyses, weight change was treated as categorical and continuous variable. Potential effect modifications of the weight change–breast cancer association were examined by (a) BMI at age 20 (dichotomised at the median BMI for premenopausal women </>21.1 kg m^−2^, and for postmenopausal women 21.3 kg m^−2^) and (b) current HRT use (yes/no in postmenopausal women only). To test for interaction, we used a metric score with median weight change within categories of weight change as the exposure variable. All tests of statistical significance were two-sided and *P*-values of <0.05 were considered statistically significant. The statistical analyses were performed with the use of the PHREG procedure in the Statistical Analysis System (SAS) software package, version 9 (SAS Institute, Cary, NC, USA).

## RESULTS

In total, 98 352 European women aged 20–80 years at baseline were followed for an average of 5.8 (±1.6) years and accrued a total of 573 102 person years. The median age at diagnosis was 49.0 years for premenopausal and 63.0 years for postmenopausal women. Baseline characteristics are presented in Table 1. Among premenopausal women, the median age at baseline varied substantially across countries due to differences in enrolment protocols between the EPIC centres. Details of other potential risk factors across weight change categories are shown in Table 2. Women with a stable weight between age 20 and study enrolment were more likely to be nulliparous, current smokers, have a university degree, and have had a higher body weight at age 20 than women who gained weight during adulthood (Table 2). On average, 26.6% of postmenopausal women used HRT at baseline. The proportion of both HRT users and OC users was smallest among women with large weight gain. The percentage of smokers was highest among women who lost weight over adulthood (<−2 kg).

### Premenopausal women

Table 3 shows the mean weight at age 20 years, adult weight change, weight, and BMI at enrolment in each of the participating countries. Weight was recalled for age 20 in all cohorts except Potsdam (Germany), where weight was recalled at age 25. Premenopausal women from the UK were the heaviest at age 20, and Italian women were the lightest. Subsequent adult weight gain was smallest for the UK women and highest for the Danish women (4.2 *vs* 12.1 kg). This large difference can be in part attributed to the different enrolment age. In all, 22% of premenopausal women had lost weight between age 20 (25) and baseline examination. The mean (s.d.) weight gain for the remaining women was 9.0±8.3 kg. At age 20, the prevalence of overweight (BMI 25–30) was 8.2% and the prevalence of obesity (BMI ⩾30) was 1.6%. At age of enrolment, these prevalences had increased to 21.0 and 8.9%, respectively.

Among premenopausal women, long-term weight gain was not associated with breast cancer risk, irrespective of adjustment for potential risk factors (Table 4). Weight loss of more than 2 kg was associated with a nonsignificant increased risk of about 50% in each of the tested models. However, this may be due to the effect of pre-existing disease, as, after excluding breast cancer cases diagnosed during the first year of follow-up, the multivariate adjusted risk estimate for weight loss approached unity (HR for <−2 kg: 1.01, CI 0.59–1.76 based on 175 cases), while relative risks in the weight gain categories were not materially changed (data not shown).

Initial weight or BMI at age 20 was inversely, but nonsignificantly associated with risk of breast cancer after adjustment for relevant covariates (data not shown), and there was no evidence of statistical interaction between BMI at age 20 and weight change in relation to breast cancer risk.

### Postmenopausal women

Similar to premenopausal women, for postmenopausal women, the heaviest women at age 20 were from the UK and the lightest were from Italy (Table 3). On average, postmenopausal women had a weight gain of 11.0 kg during adulthood, being smallest for the UK women (8.6 kg) and highest for Greek women (15.7 kg). In all, 13% of postmenopausal women had lost weight between age 20 and age at study entry. The mean (s.d.) weight gain for the remaining women was 13.5±9.5 kg. Hormone replacement therapy users had a smaller mean change in weight than those not taking hormones (10.0±9.4 and 11.7±11.4 kg, respectively). At age 20, the proportion of women who were overweight and obese was very similar to that of premenopausal women, although at the age of enrolment the prevalence of overweight (35.2%) and obesity (16.5%) in postmenopausal women was much higher than that of premenopausal women.

Among postmenopausal women overall, there was a positive trend between adult weight gain and breast cancer risk in all of the tested models, although none of the individual categorical risk estimates reached statistical significance (multivariate adjusted HR by weight change category: 0.75 (CI 0.54, 1.04); 1.0 (reference); 0.98 (CI 0.74, 1.29); 1.01 (CI 0.80, 1.28); 1.00 (CI 0.78, 1.27); 1.12 (CI 0.87, 1.45); 1.22 (CI 0.96, 1.56); trend *P*=0.002). Only after stratification by current HRT use, stronger positive associations between weight gain and breast cancer became apparent among women not currently using HRT at recruitment, and the interaction term between HRT use and weight change approached statistical significance (*P*=0.10) (Table 5). Compared to women in the reference group (±2 kg), women in the upper two categories of weight gain between 15–20 kg and >20 kg had excess risks of 50 and 52%, respectively, after adjusting for multiple covariates. Among current HRT users, weight gain was not significantly associated with breast cancer risk. Further adjustment for age at menopause (data were available for 87% of the subjects) did not substantially alter the results in nonusers or HRT users. The omission of the first year of follow-up did not substantially alter these associations (data not shown).

**Figure 1:**
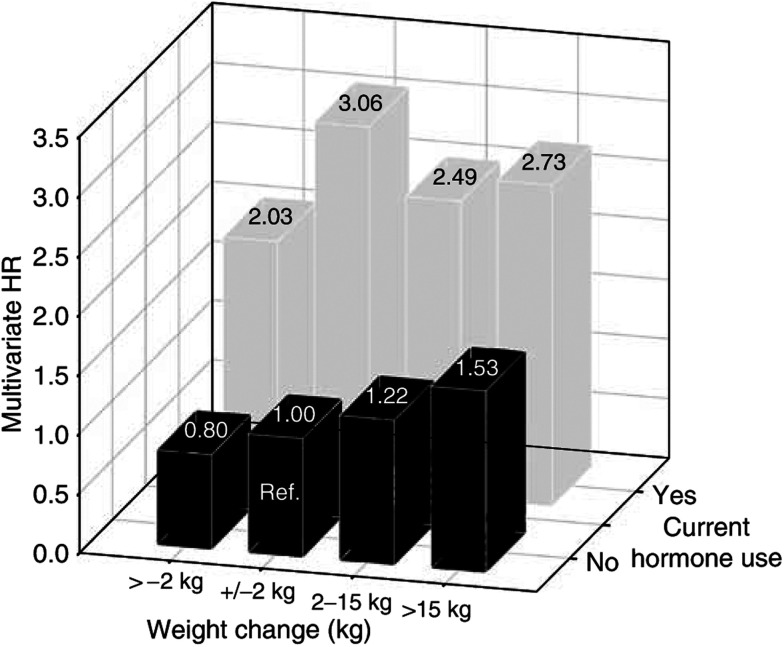
Multivariate adjusted HR of breast cancer by weight change category and current HRT use in postmenopausal women (*n*=57 923), the EPIC study.

Figure 1 shows the relative risk estimates when comparing each combined weight change–HRT use category with a uniform reference category (non-HRT user with stable weight±2 kg). Among nonusers, multivariate adjusted relative risks were increased by 22 and 53% in women gaining 2–15 kg and >15 kg, respectively, compared with women with stable weight. Overall, HRT users experienced a much higher breast cancer risk compared to non-users, irrespective of their change in weight.

Similar to premenopausal women, initial weight and BMI at age 20 were inversely, but nonsignificantly, associated with risk of postmenopausal breast cancer both before and after adjustment for weight change or other covariates (data not shown). There was no evidence of a significant interaction between BMI at age 20 (</>21.3 kg m^−2^) and weight change in relation to breast cancer risk irrespective of HRT use.

**Figure 2:**
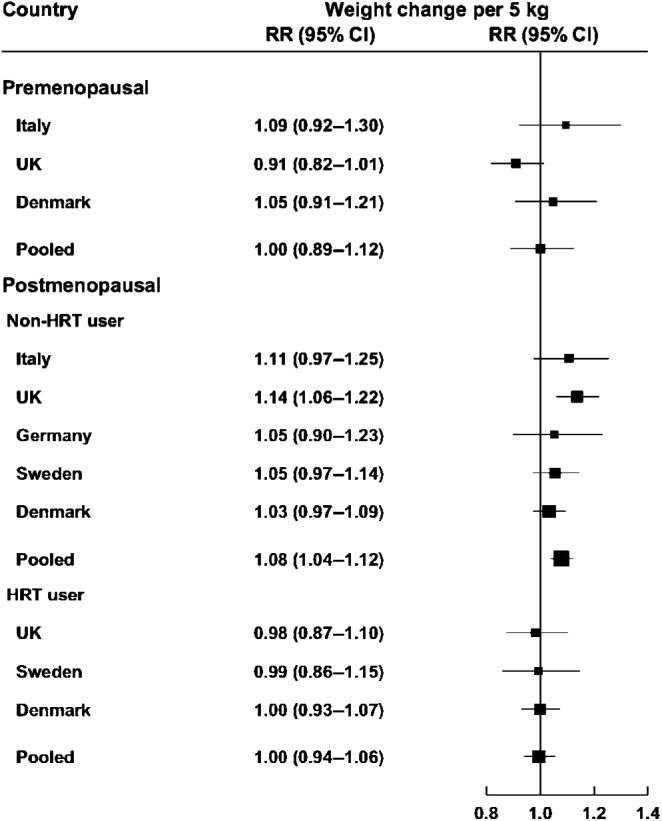
Country-specific and pooled multivariate adjusted HR of breast cancer by menopausal status and HRT use for weight change (5 kg) (country-specific risk estimates are only presented for countries with 30 or more cases), the EPIC study.

Figure 2 shows the multivariate adjusted risk estimates for breast cancer in relation to continuous weight change by 5 kg for all cohorts combined and for individual countries with at least 30 cases of breast cancer, stratified by menopausal status and HRT use. Similar to the categorical analyses, there was no association between weight change and breast cancer in premenopausal women or among postmenopausal women using HRT. Among postmenopausal women not using HRT, for one increment of weight change (5 kg), the pooled HR was 1.08 (CI 1.04–1.12), with a *P*-value for linear trend of <0.0001. No evidence of heterogeneity between countries was present for any of the presented analyses.

## DISCUSSION

In this large cohort of women from six European countries, aged 20–80 years at study enrolment, increased weight gain was significantly associated with excess breast cancer risk in postmenopausal, but not in premenopausal, women, independent of other potential risk factors.

Our finding of a nonsignificant inverse association between weight gain and premenopausal breast cancer risk is in line with evidence from the few studies that have examined this (Le Marchand *et al*, 1988; Brinton and Swanson, 1992; Huang *et al*, 1997; Coates *et al*, 1999; Peacock *et al*, 1999; Friedenreich *et al*, 2002; Weiderpass *et al*, 2004). Most of these studies report a risk reduction of about 30% for women in the highest weight change or BMI change category compared to women in the reference group with low weight gain or stable weight. However, in contrast to previous studies (Friedenreich *et al*, 2002; Weiderpass *et al*, 2004), weight loss since age 20 was not significantly associated with excess risk in the present study, specifically after excluding the first year of follow-up, which suggests that this association found in other studies may have been a reflection of pre-existing disease.

The biological mechanisms linking adiposity to premenopausal breast cancer risk are not fully understood. There is some evidence that overweight premenopausal women have more irregular and anovulatory menstrual cycles, and hence less cumulative exposure to oestrogen and progesterone, which reduces breast cancer risk (Stoll, 1997; IARC working group, 2002). Notably, the exclusion of women with reported irregular menses and/or infertility did not affect the association between adult BMI and premenopausal breast cancer risk in a Scandinavian cohort (Weiderpass *et al*, 2004), and low luteal phase serum progesterone levels were associated with higher, and not lower, breast cancer risk in an Italian cohort (Micheli *et al*, 2004). Further clarification is needed with regard to the underlying mechanisms of the inverse body size–breast cancer association before the menopause.

In postmenopausal women, the weight change–breast cancer association varied by HRT use. The examination of the modifying effect of HRT on the weight change–breast cancer association using a common reference group indicated that the increase in risk by weight gain was clearly evident in women not using HRT. Among current HRT users who have a much higher risk of developing breast cancer, there was no association between adult weight gain and cancer risk.

The effect of HRT use on the association between weight change and breast cancer risk has been previously reported (Huang *et al*, 1997; Friedenreich *et al*, 2002; Morimoto *et al*, 2002; Feigelson *et al*, 2004). The relative risk estimates in the current analysis are lower than those found in the Nurses’ Health Study among women who never used hormones (Huang *et al*, 1997), but similar to those observed in the Cancer Prevention Study (CPS)-II Nutrition cohort for comparable weight categories (Feigelson *et al*, 2004). The pooled estimates in the present study indicate that the risk per 5 kg weight gained among women not using exogenous hormones increased by 8%, which is in line with a report from a Swedish case–control study, where the excess risk per 5 kg was 7% among non-users of HRT (Magnusson *et al*, 1998).

Our finding lends support to the hypothesis that adiposity increases breast cancer risk through its oestrogenic effects (Key *et al*, 2001). Weight gain during adult life mainly reflects the deposition of fat mass rather than lean body mass. After menopause, adipose tissue is the major location for the synthesis of oestrogens from androgenic precursors (Siiteri, 1987; Bray, 2002). Accordingly, the increased risk of breast cancer in postmenopausal women who gain large amounts of weight or are overweight is attributed to excess plasma levels of oestrogens, together with low levels of sex-hormone-binding globulin (SHBG) (Stoll, 1994; Thomas *et al*, 1997; Endogenous Hormones and Breast Cancer Collaborative Group, 2003; Calle and Kaaks, 2004). Use of HRT can obscure the effect of adiposity on breast cancer risk by influencing circulating oestrogen levels; therefore, stratified analyses have been applied in recent studies. The present study found that weight change was not associated with breast cancer risk among current HRT users, which is consistent with previous studies (Harris *et al*, 1992; Huang *et al*, 1997; Magnusson *et al*, 1998; Morimoto *et al*, 2002; Feigelson *et al*, 2004) and may be attributable to the higher oestrogen levels from hormone therapy than those derived from adipose tissue (Hankinson *et al*, 1998; Trentham-Dietz *et al*, 2000). Whether the small risk reduction observed with increasing weight gain among HRT users in our study is real or due to chance finding remains to be explored in future studies.

It has been proposed that a high BMI in early adult life may influence cancer risk in later life and the potential effect modification by early body size on the association between weight change and breast cancer risk has been previously investigated (Barnes-Josiah *et al*, 1995; van der Brandt *et al*, 1997). In the present analysis, body weight at age 20 did not modify the weight change–breast cancer association in postmenopausal hormone users or non-users, which is consistent with the CPS-II Study (Feigelson *et al*, 2004).

Some limitations of these data should be noted. Our conclusions are based on results derived from a large cohort (cases *n*=1358), but with a limited follow-up period (5.8 years). Re-examination of our data after the removal of pre-existing disease by excluding individuals diagnosed during the first year of follow-up did not materially change the main findings, but did attenuate the association between weight loss and breast cancer risk among premenopausal women. Extended follow-up will allow us to evaluate the effect of excluding likely pre-existing disease with more confidence. Furthermore, it cannot be ruled out that some confounding bias is still present, due to the lack of inclusion of other unknown potential risk factors.

We were unable to evaluate in more detail the effect of weight gain at different ages, other than that of weight at age 20 and weight at enrolment. To date, only a few studies have been able to disentangle the effects of weight at different ages *vs* weight gain over a lifetime, indicating no differential effect on breast cancer risk (Trentham-Dietz *et al*, 1997; Morimoto *et al*, 2002; Radimer *et al*, 2004), or the tendency that weight gained after age 50 confers an increased risk (Friedenreich *et al*, 2002). A further limitation is that weight at age 20 was based on self-reported recall. However, evidence suggests that past body weight is reported with reasonable accuracy in comparison with measured past weight among elderly (Stevens *et al*, 1990) and middle-aged adults (Casey *et al*, 1991), even when recalled up to 30 years later. A major strength of this study is that all body measures assessed at enrolment were directly measured in a standardised way, in contrast to the self-reported data used in the majority of previous studies. Thus, nondifferential misclassification of baseline weight should be minimal.

In conclusion, these results show that large weight gain among postmenopausal women who do not use HRT is associated with an increased risk of breast cancer. Among current HRT users, who overall are at a higher risk of breast cancer than non-HRT users, weight gain is not associated with risk. These data provide further support for the positive association of adiposity with breast cancer among postmenopausal women. As obesity has reached epidemic proportions and is also a modifiable lifestyle factor, avoidance of weight gain throughout life is a salient measure for prevention of breast cancer.

## Figures and Tables

**Table 1:** Cohort characteristics by menopausal status, the EPIC study

	**Premenopausal**				**Postmenopausal**			
**Country**	**Cohort** **size (*n*)^a^**	**Baseline age** **median (range)**	**Person** **years**	**No. of** **cases^b^**	**Cohort** **size (*n*)^a^**	**Baseline age** **median (range)**	**Person** **years**	**No. of** **cases^b^**
*Italy*, Varese	2411	45 (37–57)	16 783.1	43	2945	58 (41–78)	19 997.2	65
								
*UK*	24 515	36 (20–58)	132 210.0	131	16 635	61 (39–80)	89 409.4	264
Cambridge	1510	48 (40–57)	7393.6	15	6243	63 (41–78)	32 679.8	95
Oxford – GP^c^	1373	45 (36–57)	9125.8	25	2241	57 (40–76)	14 254.3	63
Oxford – HC^d^	21 632	34 (20–58)	115 690.6	91	8151	60 (39–80)	42 675.3	106
								
*Greece*	5107	40 (20–57)	19 042.9	11	4431	61 (40–80)	16 173.7	17
								
*Germany*, Potsdam	6320	40 (20–58)	37 057.5	28	5492	59 (39–70)	33 143.1	57
								
*Sweden*, Malmo	—	—	—	—	8459	61 (45–74)	62 059.2	194
								
*Denmark*	2076	52 (50–58)	13 654.4	51	19 961	58 (50–66)	132 871.6	497
Aarhus	766	51 (50–58)	4920.5	24	5749	58 (50–66)	37 875.9	103
Copenhagen	1310	52 (50–58)	8733.9	27	14 212	58 (50–66)	95 495.7	394
								
Total	40 429	39 (20–58)	218 747.9	264	57 923	59 (39–80)	354 354.2	1094

a Women with complete measured anthropometric characteristics at enrolment and recalled weight at age 20 (German cohort: age 25).

b Invasive (malignant, primary) breast cancer.

c GP=general population.

d HC=health-conscious.

**Table 2:** Frequency distribution and mean values of covariates across weight change categories in pre- and postmenopausal women, *n*=98 352, the EPIC study

**Characteristic^a^**	**<−2 kg** **(*n*=9848)**	**±2 kg** **(*n*=14 411)**	**2–5 kg** **(*n*=13 378)**	**5–10 kg** **(*n*=21 055)**	**10–15 kg** **(*n*=15 989)**	**15–20 kg** **(*n*=10 365)**	**20+ kg** **(*n*=13 306)**
	**%**						
*Age at menarche*							
<12 years	14.6	13.6	13.2	13.6	13.3	13.1	14.6
12 years	18.4	19.5	19.5	18.9	18.6	18.5	18.6
13 years	25.9	27.3	27.0	25.1	24.6	23.7	22.7
14 years	21.0	21.5	21.5	22.7	22.8	22.7	21.1
15+ years	18.5	16.8	17.2	18.1	19.2	20.3	20.3
							
*Age at first birth*							
<20 years	5.0	4.7	5.5	7.3	8.9	10.2	12.2
20–30 years	47.9	46.0	54.5	60.9	65.8	66.5	66.5
30+ years	12.0	11.6	12.5	12.7	10.7	10.4	9.7
Nulliparous	33.6	36.2	26.2	18.6	13.7	12.1	10.5
							
*Education*							
Primary school or less	18.3	12.8	15.8	21.2	27.9	33.8	42.7
Technical school	27.3	26.6	30.4	32.2	33.0	32.2	28.8
Secondary school	15.5	18.1	14.8	14.5	14.0	12.8	11.1
University degree	30.9	35.6	32.0	24.3	17.9	14.6	11.8
							
*Alcohol consumption*							
Nondrinker	8.2	6.0	6.1	7.3	9.0	11.5	15.4
0–15 g day^−1^	76.1	77.6	77.1	74.8	73.0	71.9	70.5
15–30 g day^−1^	10.1	11.2	11.0	11.3	11.1	10.1	8.2
>30 g day^−1^	5.6	5.2	5.8	6.6	7.0	6.5	5.9
							
*Smoking status*							
Never smokers	52.1	58.9	57.0	56.1	55.1	55.5	59.1
Former smokers	22.0	21.0	23.4	24.6	25.5	25.8	24.0
Current smokers	25.2	19.2	18.9	18.4	18.5	17.6	16.0
							
*Current HRT use* b							
Yes	24.1	27.8	30.7	30.9	28.4	24.5	19.0
							
*Current oral contraceptive use* c							
Yes	16.5	21.0	18.0	14.0	9.5	7.1	3.9
							
	*Mean (s.d.)*						
Weight at age 20^d^ (kg)	64.1 (10.2)	57.5 (7.3)	56.6 (6.9)	56.1 (7.1)	55.8 (7.5)	55.7 (7.9)	56.2 (9.0)
Height, cm	163.0 (6.5)	163.4 (6.5)	163.1 (6.4)	162.6 (6.4)	162.3 (6.5)	162.2 (6.6)	162.0 (6.7)
Leisure physical activity (score)	863 (456)	840 (452)	855 (454)	865 (455)	859 (452)	859 (458)	855 (466)

a Numbers within each weight change category do not add up to 100% due to missing values.

b Postmenopausal women.

c Premenopausal women.

d Recalled weight at age 20 years, except for German cohort (recalled weight at age 25 years).

**Table 3:** Mean recalled weight at age 20 years, adult weight change, baseline weight and body mass index in 98 352 women by menopausal status and study centre, the EPIC study

	**Premenopausal (*n*=40 429) (mean (s.d.))**				**Postmenopausal (*n*=57 923) (mean (s.d.))**			
**Country**	**Weight at age** **20 years (kg)**	**Weight** **change (kg)**	**Weight (kg)**	**BMI (kg m**^−**2**^)	**Weight at age** **20 years (kg)**	**Weight change** **(kg)**	**Weight (kg)**	**BMI (kg m**^−**2**^)
*Italy*, Varese	52.8 (7.9)	9.8 (10.1)	62.6 (11.2)	24.9 (4.3)	52.6 (7.4)	12.2 (11.0)	64.8 (11.0)	26.3 (4.4)
								
*UK*	58.4 (8.6)	4.2 (8.3)	62.6 (10.9)	23.0 (3.8)	57.1 (7.8)	8.6 (10.0)	65.7 (11.4)	25.3 (4.2)
Cambridge	57.0 (7.9)	8.5 (9.1)	65.5 (12.3)	24.7 (4.4)	56.5 (7.7)	9.7 (10.0)	66.2 (11.3)	25.8 (4.2)
Oxford – GP^a^	58.0 (8.5)	8.0 (10.9)	66.0 (13.7)	24.8 (5.1)	57.0 (7.8)	9.8 (10.7)	66.8 (12.7)	25.5 (4.7)
Oxford – HC^b^	58.5 (8.7)	3.7 (7.8)	62.2 (10.5)	22.7 (3.6)	57.5 (7.8)	7.5 (9.6)	65.0 (11.1)	24.8 (4.0)
								
*Greece*	56.7 (8.1)	11.0 (11.3)	67.7 (13.0)	26.5 (5.1)	55.7 (9.9)	15.7 (13.8)	71.3 (12.3)	29.7 (5.1)
								
*Germany*, Potsdam^c^	59.4 (8.9)	6.8 (8.4)	66.2 (12.4)	24.5 (4.4)	58.9 (8.1)	11.6 (10.4)	70.5 (12.5)	27.0 (4.6)
								
*Sweden*, Malmö	—	—	—	—	55.6 (7.2)	11.7 (10.7)	67.3 (11.8)	25.4 (4.3)
								
*Denmark*	57.0 (7.4)	12.1 (10.2)	69.1 (11.9)	25.2 (4.2)	56.6 (7.6)	11.9 (10.7)	68.5 (12.1)	25.5 (4.3)
Aarhus	57.2 (7.4)	11.6 (10.1)	68.8 (11.6)	25.2 (4.1)	56.3 (7.5)	11.7 (10.7)	68.0 (11.7)	25.4 (4.2)
Copenhagen	56.9 (7.4)	12.3 (10.3)	69.2 (12.1)	25.1 (4.2)	56.7 (7.6)	12.0 (10.7)	68.7 (12.2)	25.6 (4.4)
								
Total	57.9 (8.6)	6.2 (9.4)	64.1 (11.7)	23.9 (4.3)	56.5 (7.9)	11.2 (10.9)	67.7 (12.0)	25.9 (4.5)

a GP=general population.

b HC=health-conscious.

c Recalled weight at age 25.

**Table 4:** Hazard ratio estimates of breast cancer by weight change categories in 40 429 premenopausal women, the EPIC Study

		**HR (95% CI)**		
**Weight change category**	**Number of cases**	**Model 1**	**Model 2**	**Model 3**
<−2 kg	34	1.37 (0.86, 2.18)	1.51 (0.94, 2.45)	1.56 (0.96, 2.54)
*Ref ‘±2 kg’*	37	1.00	1.00	1.00
2.1–5 kg	41	1.10 (0.70, 1.72)	1.08 (0.69, 1.69)	1.09 (0.70, 1.70)
5.1–10 kg	45	0.92 (0.61, 1.41)	0.91 (0.59, 1.38)	0.91 (0.59, 1.38)
10.1–15 kg	49	1.21 (0.78, 1.87)	1.19 (0.77, 1.85)	1.20 (0.77, 1.87)
15.1–20 kg	24	1.00 (0.59, 1.69)	0.99 (0.58, 1.67)	1.00 (0.59, 1.71)
>20 kg	24	0.85 (0.50, 1.44)	0.86 (0.51, 1.47)	0.87 (0.51, 1.49)
*P* for trend		*0.241*	*0.1732*	*0.1850*

Model 1: stratified by centre and age at recruitment.

Model 2: stratified by centre and age at recruitment, adjusted for weight at age 20.

Model 3: stratified by centre and age at recruitment, adjusted for weight at age 20, age at menarche, age at first birth/parity, OC use, education, height, alcohol intake, smoking status, and leisure physical activity.

**Table 5:** Hazard ratio estimates of breast cancer by weight change categories stratified by current HRT use in postmenopausal women, the EPIC Study

		**HR (95% CI)**		
**Weight change category**	**Number of cases**	**Model 1**	**Model 2**	**Model 3**
		*Non-HRT user (n=41 969)*		
<−2 kg	35	0.83 (0.53, 1.29)	0.79 (0.51, 1.24)	0.79 (0.50, 1.24)
*Ref ‘±2 kg’*	47	1.00	1.00	1.00
2.1–5 kg	60	1.17 (0.80, 1.72)	1.18 (0.81, 1.74)	1.19 (0.81, 1.75)
5.1–10 kg	127	1.25 (0.90, 1.76)	1.27 (0.91, 1.78)	1.27 (0.90, 1.78)
10.1–15 kg	110	1.16 (0.82, 1.64)	1.18 (0.84, 1.67)	1.18 (0.84, 1.67)
15.1–20 kg	104	1.49 (1.05, 2.10)	1.51 (1.07, 2.14)	1.50 (1.06, 2.13)
>20 kg	143	1.52 (1.09, 2.12)	1.54 (1.10, 2.15)	1.52 (1.08, 2.13)
*P* for trend		*0.0001*	*0.0001*	*0.0002*
				
		*HRT user (n=15 186)*		
<−2 kg	27	0.71 (0.44, 1.13)	0.74 (0.46, 1.19)	0.77 (0.47, 1.24)
*Ref ‘±2 kg’*	51	1.00	1.00	1.00
2.1–5 kg	51	0.79 (0.53, 1.17)	0.78 (0.53, 1.16)	0.79 (0.53, 1.17)
5.1–10 kg	106	0.80 (0.57, 1.13)	0.79 (0.57, 1.11)	0.79 (0.56, 1.10)
10.1–15 kg	97	0.86 (0.61, 1.21)	0.84 (0.59, 1.19)	0.82 (0.58, 1.17)
15.1–20 kg	56	0.79 (0.54, 1.16)	0.78 (0.53, 1.14)	0.76 (0.51, 1.13)
>20 kg	68	0.95 (0.66, 1.37)	0.94 (0.65, 1.36)	0.95 (0.65, 1.38)
*P* for trend		*0.590*	*0.7525*	*0.8660*

Model 1: stratified by centre and age at recruitment.

Model 2: stratified by centre and age at recruitment, adjusted for weight at age 20.

Model 3: stratified by centre and age at recruitment, adjusted for weight at age 20, age at menarche, age at first birth/parity, education, height, alcohol intake, smoking status, and leisure physical activity.

## References

[bib1] Ballard-Barbash R (1999) Energy Balance, Anthropometry and Cancer. Nutritional Oncology. New York: Academic Press

[bib2] Barnes-Josiah D, Potter J, Sellers T, Himes J (1995) Early body size and subsequent weight gain as predictors of breast cancer incidence (Iowa, United States). Cancer Causes Control 6: 112–118 7749050 10.1007/BF00052771

[bib3] Bray GA (2002) The underlying basis for obesity: relationship to cancer. J Nutr 132: 3451S–3455S 12421869 10.1093/jn/132.11.3451S

[bib4] Brinton LA, Swanson CA (1992) Height and weight at various ages and risk of breast cancer. Ann Epidemiol 2: 597–609 1342311 10.1016/1047-2797(92)90004-a

[bib5] Calle EE, Kaaks R (2004) Overweight, obesity and cancer: epidemiological evidence and proposed mechanisms. Nat Rev Cancer 4: 579–591 15286738 10.1038/nrc1408

[bib6] Casey V, Dwyer J, Berkey C, Coleman K, Gardner J, Valadian I (1991) Long-term memory of body weight and past weight satisfaction: a longitudinal follow-up study. Am J Clin Nutr 53: 1493–1498 2035478 10.1093/ajcn/53.6.1493

[bib7] Coates RJ, Uhler RJ, Hall HI, Potischman N, Brinton LA, Ballard-Barbash R, Gammon MD, Brogan DR, Daling JR, Malone KE, Schoenberg JB, Swanson CA (1999) Risk of breast cancer in young women in relation to body size and weight gain in adolescence and early adulthood. Br J Cancer 81: 167–174 10487629 10.1038/sj.bjc.6690667 PMC2374361

[bib8] Endogenous Hormones and Breast Cancer Collaborative Group (2003) Body mass index, serum sex hormones, and breast cancer risk in postmenopausal women. J Natl Cancer Inst 95: 1218–1226 12928347 10.1093/jnci/djg022

[bib9] Feigelson HS, Jonas CR, Teras LR, Thun MJ, Calle EE (2004) Weight gain, body mass index, hormone replacement therapy, and postmenopausal breast cancer in a large prospective study. Cancer Epidemiol Biomarkers Prev 13: 220–224 14973094 10.1158/1055-9965.epi-03-0301

[bib10] Friedenreich C (2001) Review of anthropometric factors and breast cancer risk. Eur J Cancer Prev 10: 15–32 11263588 10.1097/00008469-200102000-00003

[bib11] Friedenreich CM, Courneya KS, Bryant HE (2002) Case–control study of anthropometric measures and breast cancer risk. Int J Cancer 99: 445–452 11992416 10.1002/ijc.10389

[bib12] Haftenberger M, Lahmann PH, Panico S, Gonzales CA, Seidell JC, Boeing H, Giurdanella MC, Krogh V, Bueno-de-Mesquita HB, Peeters PHM, Skeie G, Hjartaker A, Rodriguez M, Quiros JR, Berglund GUJ, Khaw K, Spencer EA, Overvad K, Tjoennland A, Clavel-Chapelon F, Tehard B, Miller AB, Klipstein-Grobusch K, Benetou V, Kiriazi G, Riboli E, Slimani N (2002) Overweight, obesity and fat distribution in 50- to 64-year-old participants in the European Prospective Investigation into Cancer and Nutrition (EPIC). Public Health Nutr 5: 1147–1162 12639224 10.1079/PHN2002396

[bib13] Hankinson SE, Willett WC, Manson JE, Colditz GA, Hunter DJ, Spiegelman D, Barbieri RL, Speizer FE (1998) Plasma sex steroid hormone levels and risk of breast cancer in postmenopausal women. J Natl Cancer Inst 90: 1292–1299 9731736 10.1093/jnci/90.17.1292

[bib14] Harris RE, Namboodiri KK, Wynder EL (1992) Breast cancer risk: effects of estrogen replacement therapy and body mass. J Natl Cancer Inst 84: 1575–1582 1404451 10.1093/jnci/84.20.1575

[bib15] Huang Z, Hankinson S, Colditz G, Stampfer M, Hunter D, Manson J, Hennekens C, Rosner B, Speizer F, Willett W (1997) Dual effects of weight and weight gain on breast cancer risk. JAMA 278: 1407–1411 9355998

[bib16] IARC working group (2002) Weight Control and Physical Activity. Vol. 6. IARC Handbooks of Cancer Prevention. Lyon, France: IARC Press

[bib17] Key TJ, Allen NE, Verkasalo PK, Banks E (2001) Energy balance and cancer: the role of sex hormones. Proc Nutr Soc 60: 81–89 11310427 10.1079/pns200068

[bib18] Lahmann PH, Hoffmann K, Allen N, Van Gils CH, Khaw KT, Tehard B, Berrino F, Tjonneland A, Bigaard J, Olsen A, Overvad K, Clavel-Chapelon F, Nagel G, Boeing H, Trichopoulos D, Economou G, Bellos G, Palli D, Tumino R, Panico S, Sacerdote C, Krogh V, Peeters PH, Bueno-De-Mesquita HB, Lund E, Ardanaz E, Amiano P, Pera G, Quiros JR, Martinez C, Tormo MJ, Wirfalt E, Berglund G, Hallmans G, Key TJ, Reeves G, Bingham S, Norat T, Biessy C, Kaaks R, Riboli E (2004) Body size and breast cancer risk: findings from the European prospective investigation into cancer and nutrition (EPIC). Int J Cancer 111: 762–771 15252848 10.1002/ijc.20315

[bib19] Lahmann PH, Lissner L, Gullberg B, Olsson H, Berglund G (2003) A prospective study of adiposity and postmenopausal breast cancer risk: the Malmo Diet and Cancer Study. Int J Cancer 103: 246–252 12455040 10.1002/ijc.10799

[bib20] Le Marchand L, Kolonel LN, Earle ME, Mi MP (1988) Body size at different periods of life and breast cancer risk. Am J Epidemiol 128: 137–152 3381822 10.1093/oxfordjournals.aje.a114936

[bib21] Magnusson C, Baron J, Persson I, Wolk A, Bergström R, Trichopoulous D, Adami H (1998) Body size in different periods of life and breast cancer risk in post-menopausal women. Int J Cancer 76: 29–34 9533758 10.1002/(sici)1097-0215(19980330)76:1<29::aid-ijc6>3.0.co;2-#

[bib22] Micheli A, Muti P, Secreto G, Krogh V, Meneghini E, Venturelli E, Sieri S, Pala V, Berrino F (2004) Endogenous sex hormones and subsequent breast cancer in premenopausal women. Int J Cancer 112: 312–318 15352045 10.1002/ijc.20403

[bib23] Morimoto L, White E, Chen Z, Chlebowski R, Hays J, Kuller L, Lopez A, Manson J, Margolis K, Muti P, Stefanick M, McThiernan A (2002) Obesity, body size, and risk of postmenopausal breast cancer: the Women's Health Initiative (United States). Cancer Causes Control 13: 741–751 12420953 10.1023/a:1020239211145

[bib24] Peacock SL, White E, Daling JR, Voigt LF, Malone KE (1999) Relation between obesity and breast cancer in young women. Am J Epidemiol 149: 339–346 10025476 10.1093/oxfordjournals.aje.a009818

[bib25] Radimer KL, Ballard-Barbash R, Miller JS, Fay MP, Schatzkin A, Troiano R, Kreger BE, Splansky GL (2004) Weight change and the risk of late-onset breast cancer in the original Framingham cohort. Nutr Cancer 49: 7–13 15456630 10.1207/s15327914nc4901_2

[bib26] Riboli E, Hunt KJ, Slimani N, Ferrari P, Norat T, Fahey M, Charrondiere UR, Hemon B, Casagrande C, Vignat J, Overvad K, Tjoennland A, Clavel-Chapelon F, Thiebaut A, Wahrendorf J, Boeing H, Trichopoulos D, Trichopolou A, Vineis P, Palli D, Bueno-de-Mesquita HB, Peeters P, Lund E, Engeset D, Gonzales CA, Barricarte A, Berglund G, Hallmans G, Day N, Key TJ, Kaaks R, Saracci R (2002) European Prospective Investigation into Cancer and Nutrition (EPIC): study populations and data collection. Public Health Nutr 5: 1113–1124 12639222 10.1079/PHN2002394

[bib27] Riboli E, Kaaks R (1997) The EPIC project: rationale and study design. Int J Epidemiol 26: S6–S13 9126529 10.1093/ije/26.suppl_1.s6

[bib28] Siiteri PK (1987) Adipose tissue as a source of hormones. Am J Clin Nutr 45: 277–282 3541569 10.1093/ajcn/45.1.277

[bib29] Spencer EA, Appleby PN, Davey GK, Key TJ (2002) Validity of self-reported height and weight in 4808 EPIC-Oxford participants. Public Health Nutr 5: 561–565 12186665 10.1079/PHN2001322

[bib30] Stevens J, Keil J, Waid R, Gazes P (1990) Accuracy of current, 4-year, and 28-year self-reported body weight in an elderly population. Am J Epidemiol 132: 1156–1163 2260547 10.1093/oxfordjournals.aje.a115758

[bib31] Stoll B (1994) Breast cancer: the obesity connection. Br J Cancer 69: 799–801 8180007 10.1038/bjc.1994.157 PMC1968889

[bib32] Stoll B (1997) Impaired ovulation and breast cancer risk. Eur J Cancer 33: 1532–1535 9389911 10.1016/s0959-8049(97)00117-2

[bib33] Thomas H, Reeves G, Key T (1997) Endogenous estrogen and postmenopausal breast cancer: a quantitative review. Cancer Causes Control 8: 922–928 9427435 10.1023/a:1018476631561

[bib34] Trentham-Dietz A, Newcomb P, Egan K, Titus-Ernstoff L, Baron J, Storer B, Stampfer M, Willett W (2000) Weight change and risk of postmenopausal breast cancer (United States). Cancer Causes Control 11: 533–542 10880035 10.1023/a:1008961931534

[bib35] Trentham-Dietz A, Newcomb P, Storer B, Longnecker M, Baron J, Greenberg E, Willett W (1997) Body size and risk of breast cancer. Am J Epidemiol 145: 1011–1019 9169910 10.1093/oxfordjournals.aje.a009057

[bib36] van der Brandt P, Dirx M, Ronckers C, van den Hoogen P, Goldblohm R (1997) Height, weight, weight change, and postmenopausal breast cancer risk: the Netherlands Cohort Study. Cancer Causes Control 8: 39–47 9051321 10.1023/a:1018479020716

[bib37] Weiderpass E, Braaten T, Magnusson C, Kumle M, Vainio H, Lund E, Adami HO (2004) A prospective study of body size in different periods of life and risk of premenopausal breast cancer. Cancer Epidemiol Biomarkers Prev 13: 1121–1127 15247122

[bib38] Willett W (2001) Diet and breast cancer. J Intern Med 249: 395–411 11350564 10.1046/j.1365-2796.2001.00822.x

[bib39] World Health Organization (1998) Obesity: Preventing and Managing the Global Epidemic – Report of a WHO Consultation on Obesity, Geneva, 3–5 June 1997, Vol. WHO/NUT/NCD/98.1. Geneva: WHO 11234459

[bib40] Ziegler R (1997) Anthropometry and breast cancer. J Nutr 127: 924S–928S 9164265 10.1093/jn/127.5.924S

